# Tobacco smoking and alcohol drinking at diagnosis of head and neck cancer and all‐cause mortality: Results from head and neck 5000, a prospective observational cohort of people with head and neck cancer

**DOI:** 10.1002/ijc.31416

**Published:** 2018-04-23

**Authors:** Rhona A. Beynon, Samantha Lang, Sarah Schimansky, Christopher M. Penfold, Andrea Waylen, Steven J. Thomas, Michael Pawlita, Tim Waterboer, Richard M. Martin, Margaret May, Andy R. Ness

**Affiliations:** ^1^ Population Health Sciences Bristol Medical School, University of Bristol, Canynge Hall Bristol BS8 2PS United Kingdom; ^2^ MRC Integrative Epidemiology Unit (IEU) Bristol BS8 2BN United Kingdom; ^3^ National Institute for Health Research (NIHR) Bristol Biomedical Research Centre, University Hospitals Bristol NHS Foundation Trust and University of Bristol; ^4^ School of Oral and Dental Sciences, University of Bristol BS1 2LY United Kingdom; ^5^ Molecular Diagnostics of Oncogenic Infections Division German Cancer Research Center (DKFZ), Im Neuenheimer Feld 280 Heidelberg 69120 Germany; ^6^ Infections and Cancer Epidemiology German Cancer Research Center (DKFZ), Im Neuenheimer Feld 280 Heidelberg 69120 Germany

**Keywords:** head and neck cancer, smoking, alcohol, human papillomavirus, survival

## Abstract

Tobacco smoking and alcohol consumption are well‐established risk factors for head and neck cancer. The prognostic role of smoking and alcohol intake at diagnosis have been less well studied. We analysed 1,393 people prospectively enrolled into the Head and Neck 5000 study (oral cavity cancer, *n*=403; oropharyngeal cancer, *n*=660; laryngeal cancer, *n*=330) and followed up for a median of 3.5 years. The primary outcome was all‐cause mortality. We used Cox proportional hazard models to derive minimally adjusted (age and gender) and fully adjusted (age, gender, ethnicity, stage, comorbidity, body mass index, HPV status, treatment, education, deprivation index, income, marital status, and either smoking or alcohol use) mortality hazard ratios (HR) for the effects of smoking status and alcohol intake at diagnosis. Models were stratified by cancer site, stage and HPV status. The fully‐adjusted HR for current versus never‐smokers was 1.7 overall (95% confidence interval [CI] 1.1, 2.6). In stratified analyses, associations of smoking with mortality were observed for oropharyngeal and laryngeal cancers (fully adjusted HRs for current smokers: 1.8 (95% CI=0.9, 3.40 and 2.3 (95% CI=0.8, 6.4)). We found no evidence that people who drank hazardous to harmful amounts of alcohol at diagnosis had a higher mortality risk compared to non‐drinkers (HR=1.2 (95% CI=0.9, 1.6)). There was no strong evidence that HPV status or tumour stage modified the association of smoking with survival. Smoking status at the time of a head and neck cancer diagnosis influenced all‐cause mortality in models adjusted for important prognostic factors.

## Abstract 2

**What's new?**

Smoking and alcohol use are risk factors for developing head‐and‐neck cancer (HNC) and are known to influence mortality in general. However, the prognostic role of smoking status and alcohol intake at time of diagnosis on HNC survival is less clear. In this study, the authors provide a comprehensive, prospective analysis of mortality risk in different tumour sites, adjusting for important prognostic factors such as stage, comorbidity, and HPV infection. These results may provide insight to inform and improve prediction of clinical outcomes.

**Abbreviations**
BMI: body mass index
CI: confidence interval
HNC: head and neck cancer
HPV: human papillomavirus
HR: hazard ratio
HSCIC: Health and Social Care Information Centre
ICD: International Classification of Diseases
IMD: index of multiple deprivation
MIF: Median fluorescence intensity
TNM: tumor, node, metastases

Head and neck cancers (HNCs) are a heterogeneous group of tumours that arise from the mucosal epithelium of the upper aerodigestive tract. Collectively, they represent the sixth leading cause of cancer worldwide.^1^ Within the UK, HNC incidence has increased by almost a quarter in the last decade, with an estimated annual burden of ∼11,400 new cases.^2^ Since the early 1990s, oropharyngeal cancers (OPCs, tonsil and base of tongue) have seen the biggest rise of any HNC, with incidence rates more than doubling.^3^ In contrast, there has been a 20% decrease in the incidence of laryngeal cancers in the same period,^4^ though rates have levelled off more recently.^5^

Lifestyle factors play an important role in the aetiology of these cancers.^6^ Around 75% of HNCs have been attributed to the combined effects of tobacco and alcohol use.^7^ Human papillomavirus (HPV) infection, predominantly HPV‐16 infection, is also recognised as a primary risk factor for OPCs, especially in younger age groups.^8^

Despite an overall decline in HNC mortality rates,^3^ survival remains poor. The overall 5‐year survival rate is around 50%, but ranges from 33% for hypopharyngeal cancers to 60% for laryngeal cancers.^9^ People with HPV‐positive oropharyngeal tumours have consistently demonstrated improved survival compared to their HPV‐negative counterparts, despite the fact that they are frequently diagnosed at a later tumour stage.^10^ This is largely due to improved therapeutic response.^8^ People with HPV‐positive OPCs also tend to have distinct risk factor profiles, including higher socioeconomic status and a lower comorbidity,^11^ which may favour survival.

Although tobacco and alcohol drinking are responsible for the majority of new HNC cases, the prognostic role of smoking status and alcohol intake at the time of cancer presentation remains unclear, especially for people with HPV‐associated oropharyngeal tumours. In general, smoking and heavy alcohol use are both related to increased mortality risk, but estimates of the magnitude of the effects in this population are hugely inconsistent. Moreover, it has yet to be established whether smoking and alcohol use provide any additional prognostic information beyond the tumour, node, metastasis (TNM) staging system, which currently forms the basis for clinical decision making in people with HNC.

In general, the HNC literature describes a dose‐dependent increase in mortality risk with increasing exposure to tobacco pre‐diagnosis.^12^, ^13^, ^14^, ^15^, ^16^, ^17^, ^18^, ^19^ Studies have frequently been undertaken in single cancer sites, however, typically the larynx or oropharynx.^17^, ^18^, ^19^ Where studies have included multiple sites, analyses have rarely stratified on this. Both factors may help explain why estimates of the effect of smoking status on HNC survival have varied so considerably. In addition to this, studies have frequently been unable to adjust for important prognostic factors, such as comorbidity,^13^, ^14^, ^15^ body mass index^13^, ^15^, ^17^, ^18^ or HPV status,^12^, ^13^, ^14^, ^15^ often because they were conducted retrospectively.

Evidence of an association between pre‐treatment alcohol use and HNC mortality risk is conflicting. Some studies report an inverse association between alcohol intake and survival,^13^, ^19^, ^20^, ^21^ whilst others have found little or no evidence of an effect.^17^, ^22^ Consequently, it is unclear whether any association of alcohol consumption with HNC cancer mortality is genuine, or the result of residual confounding by smoking (or other factors). Recently, it was suggested that the effects of alcohol intake on HNC survival may differ by treatment method and primary site,^21^ but this study only included 427 individuals from a single cancer centre in Japan, emphasising the need for further research in this area.

An improved understanding of the prognostic significance of drinking and smoking status by site could help improve HNC outcome prediction. This could in turn help inform the lifestyle advice clinicians give to people upon a diagnosis of HNC. In the present study, we used data collected as part of a large, prospective study of over 5,500 people with HNC from across 76 UK sites (Head and Neck 5000),^23^ to examine the effect of smoking status and alcohol intake on survival in different cancer sites. To our knowledge, this is the largest study of its kind. Larger sample sizes generally lead to increased precision, and therefore our results are arguably the most accurate estimates of the effects of smoking status and alcohol intake at diagnosis on HNC mortality to date. Furthermore, given the wealth of clinical, biological and lifestyle data available, owing to the prospective study design, we were able to investigate possible interactions between smoking, alcohol and HPV status in determining mortality risk.

## Methods

### Study population

The study population included individuals enrolled in the Head and Neck 5000 clinical cohort study. Full details of the study methods and population are described in detail elsewhere.^23^ Briefly, 5,511 people with a new HNC diagnosis were recruited from 76 centres across the UK between April 2011 and December 2014. At the time of recruiting, there were approximately 180 HNC centres nationally; 78 were approached. Recruitment rates to the study varied by centre, from around 20% to around 90% of eligible HNC cases. Overall, we estimate that when all study centres were open, the study captured a third of all incident cases in the UK. Individuals were recruited before they started treatment, unless their treatment was their diagnostic procedure. At baseline, 5,474 (99%) data capture forms and 4,099 (74%) health and lifestyle questionnaires were completed.^24^ Baseline blood samples were obtained from 4,676 (85%) individuals. Full ethical approval was granted by The South West – Frenchay Regional Ethics Committee granted (ref: 10/H0107/57).

### Baseline data collection

Participants were asked to complete three self‐administered questionnaires at baseline, which included questions on social and economic circumstances, lifestyle behaviours, general health and past sexual behaviours.^23^, ^25^ Research nurses collected a blood sample from all consenting participants. Samples were frozen and stored at −80°C in the Avon Longitudinal Study of Parents and Children (ALSPAC) bio‐sample repository (http://www.bristol.ac.uk/alspac/). At each site, information on stage at diagnosis, tumour stage, treatment and various other clinical and pathologic prognostic variables was abstracted from participants’ medical records. Diagnoses were coded using the International Classification of Diseases (ICD) version 10.^26^ Clinical staging of the tumour was based on the American Head and Neck Society TNM staging of HNC.^27^

### Assessment of tobacco and alcohol exposure

Detailed information on tobacco and alcohol history was obtained at baseline *via* the self‐reported questionnaire. Participants were asked about their current smoking and drinking status and their use of tobacco and alcohol products pre‐diagnosis.^25^ Smoking status was defined as “current,” “former” or “never.” Former smokers were defined as having smoked at least one cigarette a day for a period of at least a year. Never smokers were defined as having never smoked at least one daily cigarette during a whole year. The questionnaire differentiated between use of cigarettes, hand‐rolled cigarettes, cigars and smokeless tobacco. Respondents were asked to report their average weekly alcohol consumption of a range of alcoholic beverage types before they became ill.

### Assessment of HPV status

HPV serologic testing was conducted at the German Cancer Research Center (DKFZ, Heidelberg, Germany) using glutathione S‐transferase multiplex assays.^28^ Plasma was analysed for antibodies against the HPV16 E6 oncoprotein (a marker of HPV‐transformed tumour cells^29^, ^30^), using a median fluorescence intensity (MFI) cut‐off of ≥1,000 MFI.^31^ The detection of antibodies against HPV early proteins in serum has been shown to be highly sensitive and specific for HPV16‐driven OPSCC, and consequently provides a good surrogate marker in the absence of appropriate histologic specimens.^32^

### Study follow‐up

Nurses at each site extracted up‐to‐date treatment and cancer recurrence information from participants’ medical records.^23^ All participants were flagged with NHS Digital (formerly the Health and Social Care Information Centre (HSCIC)) for ongoing notification of deaths and provision of information recorded on the death certificate.

### Statistical analysis

All analyses were performed using the data release H&N024_H&N dataset_v2.3.

#### The included population

The study involved participants with cancers of the oral cavity, oropharynx and larynx (C01‐C06; C09‐C10; C32).

#### Defining exposure

From the questionnaire data, we derived an average intake of alcohol consumption in units per week. Baseline drinking categories were then defined as none, moderate (men and women drinking <14 units/week), hazardous (men consuming 14–50 units/week; women consuming 14–35 units/week) and harmful (men consuming >50 units/week; women consuming >35 units/week), where one unit of alcohol = 8 g/10 mL ethanol.^33^ Smoking categories were consistent with those of the questionnaire (never, former and current).

#### Defining outcome

The outcome of interest was death from any cause. Follow‐up for survival analysis was defined as the time in years from study enrolment to date of death from any cause or the date of censorship (*i.e*., the last date of follow‐up). We only included all‐cause deaths in the current analysis as assignment of death as being due to HNC on death certificates is subject to misattribution bias.^34^

#### Descriptive analysis

Baseline descriptive data were stratified by tumour site and HPV status (oropharyngeal only). The *χ* ^2^‐test was used to compare the distribution of variables between groups. Kaplan–Meier survival curves were plotted to visualize survival probabilities, and differences in survival between tumour sites in relation to smoking and alcohol status were compared using the log‐rank test.

#### Missing data

Data were missing for smoking status, alcohol intake and the following covariates: body mass index (BMI), tumour stage, treatment group^1^, comorbidity^2^, ethnicity, annual household income, Index of Multiple Deprivation (IMD^3^),^35^ highest education level obtained and marital status. Missing values were imputed using the ICE package for multiple chained equations in STATA.^36^ Twenty imputed datasets were generated for each tumour site and were combined using Rubin's rule to obtain valid statistical inferences.^37^ Imputation models included the event indicator, the Nelson–Aalen estimator of the cumulative hazard,^38^ the tobacco and alcohol exposure variables, in addition to the confounders listed above.

#### Survival analysis

The primary analyses included complete cases only *i.e*. participants with complete data for confounders used in the adjusted models and information on smoking and alcohol consumption. Cox proportional hazards models, stratified by tumour site, stage and HPV status, were used to examine the associations of baseline smoking status and alcohol intake with survival. Only oropharyngeal cases were considered in the HPV stratified models because the role of HPV in tumours outside the oropharynx is uncertain, as is the ability of serology to detect HPV driven tumours in other anatomical sites. Hazard ratios (HRs) and 95% confidence intervals (CIs) for mortality were calculated for each category of smoking and drinking, using never‐smokers and non‐drinkers as the reference groups. The proportional hazard assumption was tested by plotting scaled Schoenfeld's residuals against survival time. Unadjusted Kaplan–Meier graphs were plotted to compare overall survival between cancer sites and between HPV seronegative and HPV seropositive cancers.

Minimally adjusted models included age and gender. Fully adjusted models included the following variables: clinical (tumour stage, BMI, comorbidity, treatment intent and HPV status), sociodemographic (education, annual household income, IMD and marital status, and ethnicity) and behavioural. Models evaluating the effects of smoking included adjustment for alcohol, whereas models evaluating the effects of alcohol included adjustment for smoking. To account for unobserved heterogeneity in recruitment centres, we fitted a Cox model with a shared frailty term (with gamma distribution). The significance of the frailty component was tested using a likelihood‐ratio test. To allow assessment of the effect of controlling for potential confounders, we qualitatively compared minimally adjusted with fully adjusted models.

As a sensitivity analysis, we performed the same cox regression models in the imputed dataset. Results, stratified by tumour site and HPV status can be found in Supporting Information Tables 6 and 7.

We further investigated potential interactions between: (*i*) tumour stage and smoking, (*ii*) tumour stage and alcohol consumption, (*iii*) HPV status and smoking, (*iv*) HPV status and alcohol consumption and (*v*) smoking and alcohol intake, by fitting an interaction term in the models and using a likelihood ratio test. As above, HPV analyses were restricted to the subset of participants with oropharyngeal tumours.

All reported *p*‐values are two‐sided, with *α* = 0.05. All analyses were conducted using Stata v15 (StataCorp. 2015. Stata Statistical Software: Release 14. College Station, TX: StataCorp LP).

## Results

### Baseline characteristics of study population

**Figure 1:**
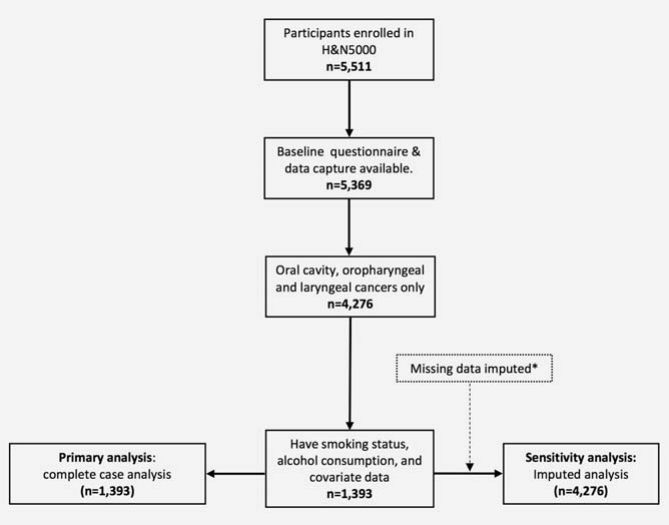
Flow of head and neck 5000 participants through the study.

The raw dataset included 5,369 individuals with HNC (Fig. 1). Of these, 4,276 had cancers of the oral cavity (*n* = 1,296), oropharynx (*n* = 1,910) and larynx (*n* = 1,070). After selecting participants with complete data, the analytic sample consisted of 1,393 individuals (oral cavity *n* = 403; oropharynx *n* = 660; larynx *n* = 330).

Descriptive characteristics of the sample, stratified by tumour site and HPV status, are presented in Tables 1 and 2 (Supporting Information Tables 1 and 2 for participants included in the imputed analysis). There were differences across tumour groups with respect to gender, age, stage, HPV status, comorbidity, smoking status, education level, annual household income, IMD quintile and marital status across tumour groups (Table 1). There was no strong evidence of a difference in the amount of alcohol consumed per week (*p* for trend = 0.739; Table 1). At the time of diagnosis, the proportion of former or current smokers was 72.7%, 70.0% and 90.6% in the oral cavity, oropharyngeal and laryngeal cancer groups, respectively. Smoking and drinking histories were comparable for participants included in the imputed analysis (Supporting Information Table 1).

**Table 1:** Baseline descriptive characteristics of participants, stratified by tumour site (*n* = 1,393)

	Oral Cavity (*n* = 403)		Oropharynx (*n* = 660)		Larynx (*n* = 330)			
Characteristic	*N*	%	*N*	%	*N*	%	Total	*p* values^a^
Survival status								N/A
Alive	288	71.50%	542	82.10%	248	75.20%	1,078	
Died	115	28.50%	118	17.90%	82	24.80%	315	
Gender								<0.001
Male	247	61.30%	539	81.70%	278	84.20%	1,064	
Female	156	38.70%	121	18.30%	52	15.80%	329	
Age group								<0.001
<50	66	16.40%	93	14.10%	17	5.20%	176	
50–64	170	42.20%	400	60.60%	125	37.90%	695	
65–79	138	34.20%	153	23.20%	159	48.20%	450	
80+	29	7.20%	14	2.10%	29	8.80%	72	
Ethnicity group								0.005
White	392	97.30%	656	99.40%	328	99.40%	1,376	
Non‐white	11	2.70%	4	0.60%	2	0.60%	17	
TNM staging								<0.001
Low	252	62.50%	93	14.10%	227	68.80%	572	
High	151	37.50%	567	85.90%	103	31.20%	821	
Serum HPV status								<0.001
Negative	392	97.30%	178	27.00%	323	97.90%	893	
Positive	11	2.70%	482	73.00%	7	2.10%	500	
Comorbidity								<0.001
None	177	43.90%	360	54.50%	138	41.80%	675	
Mild	138	34.20%	207	31.40%	111	33.60%	456	
Moderate/severe	88	21.80%	93	14.10%	81	24.50%	262	
BMI								0.002
<18.5	14	3.50%	16	2.40%	13	4.00%	43	
18.5–24.9	179	45.10%	220	33.50%	115	35.40%	514	
25–29.9	133	33.50%	275	41.90%	128	39.40%	536	
30+	71	17.90%	146	22.20%	69	21.20%	286	
Treatment group								<0.001
Surgery only	314	77.90%	59	8.90%	75	22.70%	448	
Surgery + adjunct	61	15.10%	130	19.70%	22	6.70%	213	
Chemorad only	6	1.50%	395	59.80%	49	14.80%	450	
Radio only	17	4.20%	70	10.60%	182	55.20%	269	
Palliative/supportive	5	1.20%	6	0.90%	2	0.60%	13	
Smoking								<0.001
Never	110	27.30%	198	30.00%	31	9.40%	339	
Former	197	48.90%	366	55.50%	230	69.70%	793	
Current	96	23.80%	96	14.50%	69	20.90%	261	
Alcohol consumption								0.739
Non‐drinker	119	29.50%	175	26.50%	92	27.90%	386	
Moderate	84	20.80%	156	23.60%	70	21.20%	310	
Hazardous to harmful	200	49.60%	329	49.80%	168	50.90%	697	
Highest education level								0.005
School education	181	44.90%	284	43.00%	179	54.20%	644	
College	142	35.20%	243	36.80%	110	33.30%	495	
Degree	80	19.90%	133	20.20%	41	12.40%	254	
Annual household income								<0.001
<£18.000	200	49.60%	223	33.80%	184	55.80%	607	
£18.000 to £34.999	114	28.30%	209	31.70%	94	28.50%	417	
£35.000+	89	22.10%	228	34.50%	52	15.80%	369	
IMD group								0.005
Low deprivation	184	45.70%	288	43.60%	115	34.80%	587	
Moderate deprivation	83	20.60%	155	23.50%	70	21.20%	308	
High deprivation	136	33.70%	217	32.90%	145	43.90%	498	
Marital status								0.021
Single	51	12.70%	65	9.80%	39	11.80%	155	
In a relationship	260	64.50%	485	73.50%	218	66.10%	963	
Separated/divorced/widow	92	22.80%	110	16.70%	73	22.10%	275	

*N* = number of participants.

a *p* values for trend.

**Table 2:** Baseline descriptive characteristics of participants with oropharyngeal tumours, stratified by HPV status

	HPV negative		HPV positive		
Characteristic	*N*	%	*N*	%	*p* value^a^
Survival status					<0.001
Alive	120	67.40%	422	87.60%	
Died	58	32.60%	60	12.40%	
Gender					0.149
Male	139	78.10%	400	83.00%	
Female	39	21.90%	82	17.00%	
Age group					0.339
<50	29	16.30%	64	13.30%	
50–64	98	55.10%	302	62.70%	
65–79	46	25.80%	107	22.20%	
80+	5	2.80%	9	1.90%	
Ethnicity group					0.298
White	176	98.90%	480	99.60%	
Non‐White	2	1.10%	2	0.40%	
TNM staging					<0.001
Low	43	24.20%	50	10.40%	
High	135	75.80%	432	89.60%	
Comorbidity					<0.001
None	79	44.40%	281	58.30%	
Mild	59	33.10%	148	30.70%	
Moderate/severe	40	22.50%	53	11.00%	
BMI					<0.001
<18.5	10	5.60%	6	1.30%	
18.5–24.9	81	45.50%	139	29.00%	
25–29.9	62	34.80%	213	44.50%	
30+	25	14.00%	121	25.30%	
Treatment group					0.001
Surgery only	20	11.20%	39	8.10%	
Surgery + adjunct	30	16.90%	100	20.70%	
Chemorad only	95	53.40%	300	62.20%	
Radio only	28	15.70%	42	8.70%	
Palliative/supportive	5	2.80%	1	0.20%	
Smoking					<0.001
Never	25	14.00%	173	35.90%	
Former	88	49.40%	278	57.70%	
Current	65	36.50%	31	6.40%	
Alcohol consumption					0.052
Non‐drinker	43	24.20%	132	27.40%	
Moderate	33	18.50%	123	25.50%	
Hazardous to harmful	102	57.30%	227	47.10%	
Highest education level					0.693
School education	78	43.80%	206	42.70%	
College	68	38.20%	175	36.30%	
Degree	32	18.00%	101	21.00%	
Annual household income					<0.001
<£18.000	87	48.90%	136	28.20%	
£18.000 to £34.999	51	28.70%	158	32.80%	
£35 000 +	40	22.50%	188	39.00%	
IMD group					0.555
Low deprivation	74	41.60%	214	44.40%	
Moderate deprivation	47	26.40%	108	22.40%	
High deprivation	57	32.00%	160	33.20%	
Marital status					<0.001
Single	23	12.90%	42	8.70%	
In a relationship	109	61.20%	376	78.00%	
Seperated/divorced /widow	46	25.80%	64	13.30%	

*N* = number of participants.

a *p* values for trend.

The proportion of OPCs that were HPV‐positive was 73%. People with HPV‐positive tumours were less likely to be current smokers (6.4% compared to 36.5% in the HPV‐negative group; *p* trend <0.001). There was evidence of a difference in alcohol consumption between groups (*p* trend = 0.052), with a higher proportion of hazardous to harmful drinkers in the HPV‐negative group.

### Missing data

The distribution of missing data for all participants with cancers of the oral cavity, oropharynx and larynx (*n* = 4,276), stratified by site and HPV status, are shown in Supporting Information Tables 3 and 4. Gender and age data were available for all participants. BMI data were missing for 41.9% overall (as height and weight measurements were not routinely collected at the start of the study). Smoking and alcohol data were missing for 29.0% and 30.0% of participants, respectively. Gender, age, stage, comorbidity and treatment group were comparable in people with and without missing smoking and alcohol data (Supporting Information Table 5). Those individuals with missing exposure data were more likely to live in deprived areas (high deprivation = 50.4% *vs*. 37.2% in people with smoking and alcohol data.

### Survival analysis

We found no violations of the proportionality assumption for any of the covariates included in the multivariable models. There were 315 deaths (oral cavity *n* = 115 (28.5%); oropharynx *n* = 118 (17.9%); larynx *n* = 82 (24.8%)) during a median follow‐up time of 3.5 years (25% IQR = 2.9 years; 75% IQR = 4.2 years). The proportion of deaths across tumour groups was comparable in the imputed analysis (32.9%, 24.0% and 27.7% for oral cavity, oropharyngeal and laryngeal cancers respectively). Kaplan–Meier estimates of survival by smoking and alcohol intake at diagnosis, stratified by tumour site and HPV status, are presented in Supporting Information Figures 1 and 2.

Inclusion of a frailty term did not improve the proportional hazards model (*p* values for the one‐sided likelihood ratio test = 0.13), suggesting that there is no heterogeneity in effect‐estimates by recruitment centre.

#### Smoking status and survival

For all cancer sites combined (*n* = 1,393), there was strong evidence of an association between smoking status at diagnosis and survival. Compared to never smokers, the HR for current smokers was 3.0 (95% CI = 2.1, 4.3, 3.6; *p*‐trend <0.001) in the minimally adjusted model and attenuated to 1.71 (95% CI = 1.1, 2.6; *p*‐trend = 0.001) in the fully adjusted model (Table 3). For former smokers, the HR was 1.6 (95% CI = 1.2, 2.3; *p*‐trend <0.001) in the minimally adjusted model and attenuated to 1.4 (95% CI = 1.0, 2.0; *p*‐trend 0.001) on full adjustment.

**Table 3:** Association of smoking status and alcohol intake with mortality risk, stratified by tumour site (*n* = 1,393)

	All sites (*n* = 1.393)				Oral cavity (*n* = 403)				Oropharyngeal (*n* = 660)				Larynx (*n* = 330)			
	HR	Lower CI	Upper CI	*p* value^a^	HR	Lower CI	Upper CI	*p* value^a^	HR	Lower CI	Upper CI	*p* value^a^	HR	Lower CI	Upper CI	*p* value^a^
Model 1																
Smoking				<0.001				0.036				<0.001				<0.001
Former	1.66	1.20	2.30		2.32	1.39	3.89		1.51	0.92	2.46		1.24	0.49	3.12	
Current	3.03	2.12	4.32		1.91	1.06	3.44		3.93	2.30	6.73		3.23	1.24	8.41	
Alcohol amount				0.072				0.186				0.486				0.616
Moderate	0.77	0.55	1.09		0.72	0.40	1.29		0.85	0.49	1.46		0.70	0.36	1.39	
Hazardous/harmful	1.22	0.93	1.60		1.29	0.83	2.01		1.13	0.72	1.75		1.09	0.65	1.83	
Model 2																
Smoking				<0.001				0.142				0.015				0.007
Former	1.48	1.06	1.06		2.28	1.35	3.85		1.25	0.76	2.06		1.10	0.43	2.78	
Current	1.84	1.26	2.69		1.55	0.83	2.87		2.18	1.19	3.98		2.28	0.86	6.05	
Alcohol amount				0.162				0.183				0.491				0.708
Moderate	0.77	0.54	1.08		0.76	0.42	1.38		0.86	0.49	1.49		0.62	0.31	1.24	
Hazardous/harmful	1.15	0.88	1.50		1.40	0.89	2.21		1.06	0.68	1.66		1.00	0.59	1.71	
Model 3																
Smoking				0.001				0.081				0.045				0.010
Former	1.48	1.05	1.07		2.60	1.51	4.49		1.26	0.76	2.08		1.14	0.43	3.01	
Current	1.82	1.22	1.70		1.70	0.27	3.34		1.82	0.96	3.47		2.40	0.85	6.78	
Alcohol amount				0.160				0.092				0.315				0.665
Moderate	0.79	0.56	1.12		0.79	0.43	1.45		0.95	0.54	1.67		0.63	0.31	1.26	
Hazardous harmful	1.17	0.89	1.53		1.44	0.91	2.30		1.19	0.75	1.88		1.04	0.61	1.80	
Model 4																
Smoking				0.001				0.145				0.049				0.011
Former	1.42	1.01	2.00		2.45	1.41	4.25		1.23	0.74	2.04		1.10	0.42	2.91	
Current	1.71	1.14	2.55		1.55	0.78	3 07		1.79	0.93	3.43		2.25	0.80	6.39	
Alcohol amount				0.314				0.164				0.355				0.960
Moderate	0 81	0.57	1.14		0.75	0.41	1.39		1.02	0.58	1.80		0.64	0.32	1.29	
Hazardous/harmful	1.12	0.85	1.47		1.31	0.82	2. 12		1.20	0.75	1.90		0.97	0.56	1.67	

Model 1 (minimally adjusted): adjusted for age and gender.

Model 2: additionally adjusted for clinical features (stage, treatment, comorbidity, BMI, HPV).

Model 3: additionally adjusted for social features (education, annual household income, IMD, marital status, ethnicity).

Model 4 (fully adjusted): additionally includes smoking or drinking.

HR = hazard ratio; CI = confidence interval.

a *p* values for trend.

The associations of smoking status with survival were present for oropharyngeal and laryngeal cancer groups in the subgroup analysis. In the OPC group, the HR for current smokers was 3.9 (95% CI = 2.3, 6.7; *p*‐trend <0.001) in the minimally adjusted model and 1.8 (95% CI = 0.9, 3.4; *p*‐trend 0.008) in the fully adjusted model (Table 3); for people with laryngeal cancers, the respective HRs were 3.2 (95% CI = 1.2, 8.4; *p*‐trend <0.001) and 2.3 (95% CI = 0.8, 6.4; *p*‐trend = 0.011).

Results of the imputed analysis (*n* = 4,276) were comparable to those of the complete case analysis. In minimally adjusted models, the hazard ratio was 2.9 (95% CI = 2.3, 3.7) and 1.6 (95% CI = 1.3, 1.9; *p* for trend = <0.001) for current and former smokers, respectively. In fully adjusted models, the corresponding hazard ratios were 1.6 (95% CI = 1.2, 2.1) and 1.3 (95% CI = 1.1, 1.6; *p* for trend = <0.001) (Supporting Information Table 6). A similar pattern of association was seen in the stratified analysis.

#### Alcohol intake and survival

In the minimally adjusted model, the hazard ratio for hazardous to harmful drinkers compared to non‐drinkers was 1.2 (95% CI = 0.9, 1.6; *p*‐trend = 0.072) (Table 3). On full adjustment, there was no evidence for an increase in mortality risk (HR = 1.1 (95% CI = 0.9, 1.5; *p*‐trend = 0.314). There was weak evidence to suggest that moderate drinkers experienced improved survival compared to non‐drinkers in the minimally adjusted model (HR = 0.8, 0.6, 1.1; *p* for trend = 0.072), but this association was not robust to adjustment.

When models were stratified by tumour site, there was no evidence of an association between alcohol consumption and survival.

In the imputed analyses, a comparable pattern of association between alcohol drinking and survival was observed (minimally adjusted model: HR = 1.2 (95% CI= 1.0, 1.4) and 0.9 (95% CI = 0.7, 1.1; *p* for trend = <0.036) for hazardous/harmful and moderate drinkers, respectively, fully adjusted: HR = 1.1 (95% CI = 0.9, 1.3) and 0.9 (95% CI = 0.7, 1.1) (Supporting Information Table 6).

#### Influence of tumour stage on the associations of smoking and alcohol intake with survival

There were 572 people with low stage tumours and 821 with high stage tumours in the analysis. On full adjustment, the hazard of death for current versus never‐smokers was 2.3 in the low stage group (95% CI = 1.1, 4.9; *p* for trend = 0.075), and 1.7 in the high stage group (95% CI = 1.0, 2.8; *p* for trend = <0.004) (Table 4). There was no evidence of an association between the amount of alcohol consumed at diagnosis and survival in either the low or high stage subgroups.

**Table 4:** Association of smoking status and alcohol intake with mortality risk, stratified by tumour stage (*n* = 1,393)

	Low stage (*n* = 572)				High stage (*n* = 821)			
	HR	Lower CI	Upper CI	*p* value^a^	HR	Lower CI	Upper CI	*p* value^a^
Model 1								
Smoking								<0.001
Former	2.02	1.05	3.86		1.65	1.13	2.41	
Current	2.90	1.44	5.82	0.002	3.50	2.31	5.29	
Alcohol amount								0.445
Moderate	0.67	0.35	1.30		0.75	0.50	1.12	
Hazardous/harmful	1.45	0.90	2.34	0.074	1.08	0.78	1.50	
Model 2								
Smoking								0.005
Former	2.05	1.06	3.97		1.35	0.92	1.99	
Current	2.38	1.16	4.90	0.052	1.75	1.11	2.75	
Alcohol amount								0.619
Moderate	0.72	0.37	1.40		0.80	0.53	1.19	
Hazardous/harmful	1.50	0.92	2.45	0.121	1.05	0.76	1.45	
Model 3								
Smoking								0.004
Former	2.15	1.09	4 23		1.40	0.94	2.09	
Current	2.54	1.19	5.45	0.075	1.77	1.09	2.86	
Alcohol amount								0.655
Moderate	0.71	0.36	1.40		0.81	0.54	1.23	
Hazardous/harmful	1.58	0.96	2.60	0.086	1.04	0.75	1.46	
Model 4								
Smoking								0.004
Former	2.00	1.01	3.97		1.37	0.92	2.05	
Current	2.29	1.06	4.94	0.075	1.70	1.04	2.77	
Alcohol amount								0.904
Moderate	0.68	0.34	1.33		0.85	0.56	1.29	
Hazardous/harmful	1.44	0.88	2.36	0.135	1.02	0.73	1.42	

Model 1 (minimally adjusted): adjusted for age and gender.

Model 2: additionally adjusted for clinical features (stage, treatment, comorbidity, BMI).

Model 3: additionally adjusted for social features (education, annual household income, IMD, marital status, ethnicity).

Model 4 (fully adjusted): additionally includes smoking or drinking.

HR = hazard ratio; CI = confidence interval.

a *p* values for trend.

In the imputed analysis, there was a 2.2‐fold higher mortality risk (95% CI = 1.4, 3.6; *p*‐trend = <0.001) for current versus never smokers in the low stage tumour group (*n* = 1,701) compared to a 1.4‐fold higher mortality risk (95% CI = 1.1, 2.0; *p* < 0.007) in the high stage tumour group (*n* = 2,562). The HRs for hazardous to harmful drinkers were similar to those in the primary analyses (Supporting Information Table 7).

#### Influence of HPV status on the associations of smoking and alcohol intake with survival

Results of the HPV‐stratified analyses are presented in Table 5. In fully adjusted models (*n* = 660), the HR for current versus never smokers was 1.3 (95% CI = 0.3, 5.1; *p*‐trend = 0.481) in the HPV‐negative group (*n* = 178) and 2.1 (95% CI = 0.8, 5.3; *p*‐trend = 0.263) in the HPV‐positive group (*n* = 482). The mortality hazards for hazardous to harmful drinkers versus non‐drinkers were 2.6 (95% CI = 1.2, 5.6; *p*‐trend = 0.012) and 0.6 (95% CI = 0.3, 1.1; *p*‐trend = 0.160) in HPV‐negative and HPV‐positive groups, respectively. Results of the imputed analyses (*n* = 1,595) are presented in Supporting Information Table 8. Here, there was evidence that current smokers were at increased risk of death compared to non‐smokers, regardless of HPV status (fully adjusted HR = 1.9 (95% CI = 0.9, 4.1; *p* for trend = 0.070) and 2.0 (95% CI = 1.0, 4.0; *p* for trend = 0.049) for HPV‐negative and HPV‐positive groups, respectively).

**Table 5:** Association of smoking status and alcohol intake with mortality risk, stratified by HPV status (*n* = 660)

	HPV negative (*n* = 178)				HPV positive (*n* = 482)			
	HR	Lower CI	Upper CI	*p* value^a^	HR	Lower CI	Upper CI	*p* value^a^
Model 1								
Smoking				0.002				0.313
Former	3.02	0.91	10.05		1.08	0.61	1.89	
Current	5.12	1.53	17.18		1.82	0.75	4.42	
Alcohol amount				0.038				0.096
Moderate	0.70	0.26	1.90		0.89	0.46	1.71	
Hazardous/harmful	1.84	0.92	3.65		0.60	0.33	1.12	
Model 2								
Smoking				0.188				0.271
Former	2.16	0.64	7.23		1.05	0.59	1.87	
Current	2.58	0.75	8.91		1.88	0.77	4.61	
Alcohol amount				0.016				0.093
Moderate	0.76	0.26	2.24		0.93	0.48	1.79	
Hazardous/harmful	2.01	1.00	4.03		0.59	0.32	1.09	
Model 3								
Smoking				0.493				0.265
Former	2.30	0.67	7.96		1.03	0.57	1.85	
Current	1.71	0.45	6.50		2.02	0.80	5.10	
Alcohol amount				0.008				0.119
Moderate	1.19	0.38	3.76		0.90	0.46	1.77	
Hazardous/harmful	2.82	1.34	5.91		0.57	0.30	1.11	
Model 4								
Smoking				0.481				0.263
Former	1.53	0.43	5.48		1.07	0.59	1.94	
Current	1.30	0.33	5.14		2.06	0.81	5.25	
Alcohol amount				0.012				0.160
Moderate	1.11	0.34	3.59		0.93	0.47	1.83	
Hazardous/harmful	2.59	1.20	5.61		0.58	0.30	1.12	

Model 1 (minimally adjusted): adjusted for age and gender.

Model 2: additionally adjusted for clinical features (stage, treatment, comorbidity, BMI).

Model 3: additionally adjusted for social features (education, annual household income, IMD, marital status, ethnicity).

Model 4 (fully adjusted): additionally includes smoking or drinking.

HR = hazard ratio; CI = confidence interval.

a *p* values for trend.

There was no strong evidence that HPV status modified the association of smoking with survival in the sensitivity analyses (*p*‐interaction = 0.563). The effect of alcohol may differ by HPV status (*p*‐interaction = 0.024), but this may be due to chance as the number of deaths was small (58 in the HPV negative group and 60 in the HPV positive group).

#### Interaction of tobacco and alcohol

We found no evidence of an interaction between smoking and alcohol consumption on survival (*p*‐interaction = 0.233 in the fully adjusted model).

## Discussion

### Principle findings

The major finding of this large, prospective study is that, even after adjusting for a wide‐range of prognostic factors (confounders), smoking status at the time of a HNC diagnosis is associated with worse survival. In fully adjusted models, current smokers had around a 70% higher all‐cause mortality risk compared to people who had never smoked, whilst former smokers were over 40% more likely to die during follow‐up. Drinking behaviour around the time of diagnosis was not associated with overall mortality risk in this analysis.

Our findings are in line with those of earlier studies, which suggest that smoking at the time of a HNC diagnosis may result in poorer clinical outcomes and reduced survival.^12^, ^13^, ^15^, ^39^ Estimates of the size of the effect have varied considerably however, ranging from a 2.4‐fold higher all‐cause mortality risk in current versus never‐smokers^12^ to an almost fivefold higher mortality risk in people with >60 pack‐years of smoking versus never‐smokers.^13^ There are a number of possible explanations for this. First, much of the evidence is based on retrospective analyses of population‐level cancer registries, which are often incomplete or incorrect.^40^ Consequently, studies have frequently been missing information on important clinical and lifestyle factors such as comorbidity, BMI and socioeconomic position, which could potentially confound the association of smoking with survival. Those studies which have been conducted prospectively are small—typically five hundred persons or fewer,^12^, ^13^, ^14^ and as a result they have limited statistical power to detect an accurate measure of the effect. Second, estimates have been derived from different subpopulations of people, *i.e*. different HNC sites or tumour stages, which are often not considered separately. This could bias estimates of the effect of smoking on survival if mortality risk is greater or lesser in certain tumour groups.

With respect to alcohol consumption, the existing literature is limited and conflicting. Our results support those of Duffy *et al*. who found no difference in mortality risk between active drinkers and non‐drinkers after adjusting for a wider range of confounders.^12^ In contrast to this however, Mayne *et al*. reported a fivefold increased mortality risk for persons who drank >35 drinks per week compared to those who abstained.^13^ Both studies were relatively small (504 and 204 people, respectively), and enrolled participants from either a single centre^12^ or a single clinical trial,^13^ limiting their generalisability.

It is biologically plausible that, as well as being risk factors for HNC, smoking could reduce survival following a diagnosis. One way in which smoking could influence survival is through its effects on treatment response. A growing body of evidence suggests that smokers have an increased risk of treatment‐related adverse events and poorer clinical outcomes following radiotherapy, compared to non‐smokers.^41^, ^42^ The biological mechanisms underpinning this association are not fully understood, but increased tumour hypoxia, resulting from increased carboxyhaemoglobin (the binding of carbon monoxide to haemoglobin) in smokers, is a likely explanation.^43^ In addition to this, it has been suggested that tobacco reduces the efficacy of radiotherapy through triggering a p53 mutation that could promote resistance to apoptosis.^44^ Smoking is also known to effect inflammatory responses^45^ and immune competence,^46^ which could increase the likelihood of adverse clinical outcomes.

### Strengths and limitations of the study

This study has several strengths. These include the prospective population‐based cohort design, the relatively large sample size and our ability to adjust for multiple biological, clinical and lifestyle covariates, including HPV. In addition to this, we explored the risk of bias due to missing data by employing a multiple imputation approach.^47^ Results of the imputed analysis was broadly consistent with those of the complete case analysis.

The study has several limitations. First, as in most previous studies, assessments of smoking and alcohol intake were based on participants’ self‐reports. Prior work has shown that self‐reports can often provide an inaccurate assessment of tobacco and alcohol use, particularly in people who have recently been diagnosed with cancer.^48^ This would most likely result in an underestimation of the effects of smoking and drinking on HNC survival.

Second, although we adjusted for several confounders in our models, residual confounding by unmeasured or poorly measured factors, such as a delay in receiving treatment or other lifestyle factors related to smoking and drinking behaviours (*e.g*., physical activity and dietary intake), is possible. Furthermore, it is possible that the individuals included in the complete case analysis were different to those in the imputed analysis, since those with missing smoking and alcohol data lived in more deprived areas overall. However, given that results were comparable using both approaches, it seems unlikely that the complete case analysis was biased by socioeconomic position.

Third, whilst the sample size was sufficient for us to detect the main effects of baseline smoking status and alcohol intake on survival, it was insufficient to examine interactions between these two exposures and HPV in determining mortality; it also limited our ability to investigate whether the effects of smoking and drinking on survival were modified by cancer site. This was because there were only a small number of events (deaths) in each subgroup. We had no prior hypotheses that smoking or alcohol intake would have a greater or lesser effect on survival in any one cancer group however, and therefore the analyses were exploratory by design.

Fourth, we used HPV‐specific antibody levels to identify individuals with HPV‐positive oropharyngeal tumours, but the presence of HPV16 DNA is considered the gold standard measure. Previous studies have confirmed, however, that detection of antibodies against E6 and E7 oncoproteins shows good correlation with HPV DNA in the tumour tissue.^32^ Kreimer *et al*. showed that high HPV viral load increased the odds of HPV16 E6 seropositivity 57‐fold and HPV16 E7 seropositivity 26‐fold. Moreover, HPV16 E6 antibodies have also been shown to be independent favourable prognostic factors in OPCs.^49^, ^50^ Some HPV‐related OPCs are mediated by other HPV genotypes, including HPV18 (1–8% of OPCs), and less commonly HPV33, −35, −56 and −67.^51^ We only considered HPV16 in the current analysis because 69% of the 1,910 participants with OPCs were HPV16 positive, compared to only 2% who were HPV18 positive.

Finally, we were unable to examine whether baseline smoking status and alcohol intake influenced cancer‐specific mortality as cause‐specific mortality data were not available for all participants. Previous studies suggest that death from non‐cancerous causes (competing mortality) and second primary malignancies are important events in HNC^52^ and could provide greater insight into the biological mechanisms that underlie the associations of smoking and drinking with survival. The cause of death information on a death certificate is often inaccurate however.^53^ Accuracy of all‐cause mortality is solely dependent on the number of deaths identified, and is arguably a more reliable outcome measure.

### Policy implications

Our results emphasise the importance of clinicians recording information on peoples’ smoking status in their medical notes at the time of diagnosis, to help identify those at risk of poor survival. It is possible that smoking cessation and reduced alcohol consumption could reduce mortality in this population, which highlights the need to take advantage of the “teachable moment” that a cancer diagnosis presents.^54^ Clinicians routinely advise people with HNC to stop smoking, but our data provides an impetus for health providers and policy makers to ensure that this remains a focus of care.

### Future research

There remain several unanswered questions concerning the role of smoking status and alcohol use in HNC survival. First, it is unclear whether the associations of smoking and alcohol use at the time of diagnosis with survival vary in different tumour sites. In the current study, the mortality hazard for current smokers was much lower in the oral cavity cancer group than it was in the oropharyngeal and laryngeal cancer groups, suggesting that tumour site may modify the relationship between smoking status and survival. This finding needs to be validated in other HNC cohorts.

The second unanswered question is, what influence does HPV have on the associations of tobacco and alcohol use with survival? It has been shown that smoking increases the risk of OPC, irrespective of HPV16 status,^55^ but evidence of an effect of joint exposure on survival after diagnosis is conflicting. Some studies report reduced survival in HPV‐positive smokers compared to their non‐smoking counterparts,^18^ whilst others report no difference in prognosis between HPV‐positive smokers and HPV‐positive non‐smokers.^56^ To the best of our knowledge, no studies have compared the prognostic value of alcohol use in people with HPV‐positive versus HPV‐negative OPCs. To adequately address these questions, further well‐designed studies in people with oropharyngeal tumours are needed, which are large enough to ensure that there is adequate power to detect an interaction. Furthermore, HPV testing needs to be included, where possible, in all future studies involving OPC cases.

The final question is whether survival can be improved with smoking cessation and reduced alcohol consumption after diagnosis. Given that at least one‐third of people with HNC continue to smoke and 16% continue to drink hazardously^57^ after receiving the primary HNC diagnosis, this is an important question to address. Few studies in the literature have examined patterns of smoking and alcohol drinking after diagnosis, but there is some evidence to suggest benefits of smoking cessation in terms of response to radiation therapy and reduced risk of second primary tumours.^58^ If smoking cessation and reduced alcohol consumption can improve survival rates in this population, then pilot behavioural intervention trials should be conducted to identify the most effective way of supporting individuals to make and sustain changes.

An issue which is pertinent to all future research in this area is the accurate measurement of smoking and alcohol use in observational studies. As highlighted above, self‐reported intake is frequently unreliable, but recent advances in genome‐wide methylation profiling have permitted the identification of robust biomarkers of tobacco and alcohol exposure,^59^, ^60^ circumventing issues of reporting bias. Another approach that could be used to obtain unbiased estimates of the causal effects of tobacco and alcohol consumption on HNC survival is Mendelian Randomization, whereby genetic variants which modulate an observed risk factor are used as a proxy measure for that exposure, eliminating problems of confounding and reverse causation.^61^

## Conclusions

In this large prospective clinical cohort of people with HNC we showed that smoking status at the time of diagnosis is associated with poorer survival. Further research should explore whether interventions that encourage smoking cessation or reduced alcohol consumption result in improved outcomes.

## Supporting information

Supporting Information 1

Supporting Information 2

Supporting Information 3

## Footnotes

1 Refers to the intent of the cancer care plan for the patient's head and neck cancer at the point of diagnosis e.g. radiotherapy or chemotherapy.

2 Comorbidity was measured using the Adult Co‐morbidity Evaluation (ACE‐27) assessment tool. Categories were defined as “mild‐“, “moderate”‐, and “severe decompensation”,

3 IMD is the official measure of relative deprivation ranks every small area (or neighbourhood) in England from 1 (most deprived area) to 32,844 (least deprived area).
