# Supplementary material for: Tobacco smoking and alcohol drinking at diagnosis of head and neck cancer and all‐cause mortality: Results from head and neck 5000, a prospective observational cohort of people with head and neck cancer
Source: Int J Cancer. 2018 Apr 23;143(5):1114–27. doi: 10.1002/ijc.31416 (PMC6099366; doi:10.1002/ijc.31416)
Supplement: Supplementary file 3 — Supporting Information 3 [file IJC-143-1114-s003.docx]

**Supplementary table 1**: Baseline descriptive statistics for participants included in the MI analysis (n=4,276).

N= number of participants. *p-value for trend.

**Supplementary table 2**: Baseline descriptive statistics for participants included in the MI analysis, stratified by HPV status (n=1,595).

HPV serology data was missing for n=315 (OP total n=1,910).

**Supplementary table 3**: Distribution of missing data, stratified by tumour site.

.

N= number of participants.

**Supplementary table 4**: Distribution of missing data, stratified by HPV status (oropharyngeal cases only n=1,595).

N= number of participants.

HPV serology data was missing for n=315 (OP total n=1,910).

**Supplementary table 5:** Comparison of baseline descriptive characteristics for participants with and without smoking and alcohol data.

N= number of participants.

*p-value for trend.

**Supplementary table 6**: Results of the multiple imputation analysis, stratified by tumour site.

Model 1 (minimally adjusted): adjusted for age and gender;

Model 2: additionally adjusted for clinical features (stage, treatment, comorbidity, bmi, HPV);

Model 3: additionally adjusted for social features (education, annual household income, IMD, marital status, ethnicity);

Model 4 (fully adjusted): additionally includes smoking or drinking.

HR= hazard ratio; CI= confidence interval. *p-value for trend.

**Supplementary table 7:** Results of the multiple imputation analysis, stratified by tumour stage.

Model 1 (minimally adjusted): adjusted for age and gender;

Model 2: additionally adjusted for clinical features (stage, treatment, comorbidity, bmi, HPV);

Model 3: additionally adjusted for social features (education, annual household income, IMD, marital status, ethnicity);

Model 4 (fully adjusted): additionally includes smoking or drinking.

HR= hazard ratio; CI= confidence interval.

*p-value for trend.

Tumour stage was imputed for 13 participants.

**Supplementary table 8:** Results of the multiple imputation analysis, stratified by HPV status (oropharyngeal cases only).

Model 1 (minimally adjusted): adjusted for age and gender;

Model 2: additionally adjusted for clinical features (stage, treatment, comorbidity, bmi, HPV);

Model 3: additionally adjusted for social features (education, annual household income, IMD, marital status, ethnicity);

Model 4 (fully adjusted): additionally includes smoking or drinking.

HR= hazard ratio; CI= confidence interval.

*p-value for trend
